# Evidence for a Lectin Specific for Sulfated Glycans in the Salivary Gland of the Malaria Vector, *Anopheles gambiae*

**DOI:** 10.1371/journal.pone.0107295

**Published:** 2014-09-10

**Authors:** Ivo M. B. Francischetti, Dongying Ma, John F. Andersen, José M. C. Ribeiro

**Affiliations:** Section of Vector Biology, Laboratory of Malaria and Vector Research, National Institute of Allergy and Infectious Diseases, National Institutes of Health, Bethesda, Maryland, United States of America; Kansas State University, United States of America

## Abstract

Salivary gland homogenate (SGH) from the female mosquitoes *Anopheles gambiae*, *An. stephensi*, *An. freeborni*, *An. dirus* and *An. albimanus* were found to exhibit hemagglutinating (lectin) activity. Lectin activity was not found for male *An. gambiae*, or female *Ae aegypti*, *Culex quinquefasciatus*, *Phlebotomus duboscqi*, and *Lutzomyia longipalpis*. With respect to species-specificity, *An. gambiae* SGH agglutinates red blood cells (RBC) from humans, horse, sheep, goat, pig, and cow; it is less active for rats RBC, and not detectable for guinea-pigs or chicken RBC. Notably, lectin activity was inhibited by low concentrations of dextran sulfate 50–500 K, fucoidan, heparin, laminin, heparin sulfate proteoglycan, sialyl-containing glycans (*e.g.* 3′-sialyl Lewis X, and 6′-sialyl lactose), and gangliosides (*e.g.* GM3, GD1, GD1b, GTB1, GM1, GQ1B), but not by simple sugars. These results imply that molecule(s) in the salivary gland target sulfated glycans. SGH from *An. gambiae* was also found to promote agglutination of HL-60 cells which are rich in sialyl Lewis X, a glycan that decorates PSGL-1, the neutrophils receptor that interacts with endothelial cell P-selectin. Accordingly, SGH interferes with HL-60 cells adhesion to immobilized P-selectin. Because *An. gambiae* SGH expresses galectins, one member of this family (herein named Agalectin) was expressed in *E. coli*. Recombinant Agalectin behaves as a non-covalent homodimer. It does not display lectin activity, and does not interact with 500 candidates tested in a Glycan microarray. Gel-filtration chromatography of the SGH of *An. gambiae* identified a fraction with hemagglutinating activity, which was analyzed by 1D PAGE followed by in-gel tryptic digestion, and nano-LC MS/MS. This approach identified several genes which emerge as candidates for a lectin targeting sulfated glycans, the first with this selectivity to be reported in the SGH of a blood-sucking arthropod. The role of salivary molecules (sialogenins) with lectin activity is discussed in the context of inflammation, and parasite-vector-host interactions.

## Introduction

Salivary glands of blood-sucking mosquitoes, sand flies, and ticks play a critical role in feeding [1]. In mosquitoes, the mouth parts penetrate the skin and canulate arterioles or venules, and blood is ingested. Blood is also collected from hemorrhagic pools, as recently visualized by intravital video microscopy [2], [3]. During this process, saliva is released, and several salivary components counteract the host response triggered by the injury caused by bites [4], [5]. For example, mosquitoes express several antihemostatics including anophelin that targets thrombin [6], apyrases which account for inhibition of ADP-induced platelet aggregation [7], [8], antigen-5 family members that exhibit metal-dependent antioxidant activity [9] and a myeloperoxidase that promotes vasodilation [10].

Salivary gland homogenate (SGH) from Anophelinae and other blood-sucking arthropods also exhibits lectin activity. For example, Nayar and Knight and other investigators demonstrated hemagglutinating activity for *Anopheles quadrimaculatus*, against RBC from humans, mule, cows, pig, dog, rabbit, guinea-pig, rat and mouse [11]–[13]. *Culex molestus* and *An. maculipennis* also promote hemagglutination of RBC from human, donkey, rabbit, and dog while *Aedes angustivitatus* agglutinates human O, A and B erythrocytes. *Ae aegypti*, *Ae. cinereus*, *Ae. atropalpus*, *Ae. punctor*, *Ae. fitchii*, *Culiseta inornata*, and *Culex pipiens* agglutinate rabbit erythrocytes [12]–[15]. Surprisingly, salivary lectins have not been molecularly characterized. Identification of these components probably lays in the transcriptome and proteome (sialome) analysis of the glands, which characteristically contain several cDNAs coding for members of the C-type lectin and galectins super families [7], [16].

Given the identification of sequences compatible with lectins in the sialome of *Anopheles* spp. [7], [8], [17], the aim of this study has been to investigate further this activity. We revealed that the hemagglutinating activity of the SGH of *An. gambiae* is inhibited by sulfated glycans. We have also identified several molecules that emerge as candidates to molecularly characterize *An. gambiae* SGH lectin activity.

## Materials and Methods

### Materials

Dextran sulfate (DS) 5,000, DS 50,000, DS 500,000, dextran, heparin grade II, condroitin sulfate A, and C, heparan sulfate, keratan sulfate, glycophorin, asialoglycophorin, orosomucoid, fetuin, asialofetuin, laminin, heparin sulfate proteoglycan, maltose, galactose, trehalose, manose, glucose and N-acetyl-galactosamine, and sialic acid were from Sigma Chemical Co. (Saint Louis, MO). 3′-sialyl Lewis X, 3′-sialyl Lewis A, 3′-sulfated Lewis A, 3′-sialyl lactose, 6′-sialyl lactose, tetrasacharide -A, -B, and -C, di-sialyl lacto-tetraose, 3′-sialyl fucosil lactose, monosialoganglioside (GM3), disialoganglioside (GD1a), disialoganglioside (GD1b), trisialoganglioside (GT1B), tetrasialoganglioside (GM1), and tetrasialoganglioside (GQ1B) were from Oxford GlycoSytems (Oxford, UK) or from Carbosynth (Berkshire, UK). Other reagents were from Sigma Chemical Co (Saint Louis, MO).

### Mosquitoes

*Anopheles gambiae, An. stephensi, An. albimanus, An. freeborni, An. dirus, Culex quinquefasciatus, Lutozomyia longipalpis*, and *Aedes aegypti* were reared at the NIAID/Laboratory of Malaria and Vector Research mosquito facility under the supervision of Mr. André Laughinhouse. Salivary glands were dissected under the microscope, placed in PBS (30 pairs/30 µl) and frozen at −80°C. When required, the tubes were sonicated in a Misonix 3000 sonicator (Farmingdale, NY) and centrifuged for 5 min at 13,000×g. The supernatant was collected (herein named salivary gland homogenate, SGH) and frozen at −80°C.

### Blood collection and ethics statement

Blood samples (human blood type O) were obtained from paid healthy volunteers who gave written informed consent to participate in an Institutional Review Board (IRB) approved protocol under the name “Collection and Distribution of Blood Components from Healthy Donors for In Vitro Research Use”. The protocol is designed to protect subjects from research risks as defined in 45CFR46 and to abide by all NIH guidelines for human subjects research (protocol number 99-CC-0168). Collection was performed at the NIH Department of Transfusion Medicine under the direction of Dr. Susan Leitman, as described [18].

### Hemagglutinating activity

Human blood (O type) was collected in the presence of citrate (0.33% v/v, final concentrations) or EDTA (5 mM), and centrifuged at 2000×g for 10 min. The supernatant containing plasma, and the buffy coat were discarded and red blood cells were diluted in Hepes-saline pH 7.4, and washed 3 times. Then, RBCs were re-suspended to 4% in Hepes-saline supplemented with BSA (0.1% *v/v*; PBS-BSA). In some experiments CaCl_2_ (5 mM) was added to the buffer. For hemagglutinating activity, SGH was serially diluted in 50 µl of PBS-BSA in a 96-well round bottom Costar microplate (Corning Inc. NY). Then, 50 µl of a red blood cell suspension (in Hepes-saline-BSA) was added, plates briefly agitated, and left in the bench at room temperature. Results were read after one hour, and the presence of red blood cell pellet, like a red dot, indicated no hemagglutination. The ratio 1/*n*, where *n* is the maximum dilution of pair of glands required for hemagglutination was used to estimate the specific activity of each gland. For example, a gland that promoted visible agglutination with 0.25 pair/assay exhibits specific activity of 4 U/assay (or 1/0.25).

### Cloning and expression of Agalectin in *E. coli*

Synthetic cDNA for Agalectin (gi 118795154 and gi347965476) was produced by Biobasics (Ontario, CA). The sequence displays an N-terminal *NdeI* and a C-terminal *XhoI* restriction site. The *NdeI* site adds a 5′-methionine codon to all sequences that acts as start codon in the bacterial expression system, whereas the *XhoI* site was incorporated after the stop codon. pET 17b constructs were confirmed before transformation of *Escherichia coli* strain BL21 (DE3) pLysS cells (Invitrogen) which were plated in ampicillin-Agar plates (K&D Biomedicals). For recombinant protein production, 30 mL of Luria Bertani broth (with added 37 µg/ml chloramphenicol and 100 µg/ml ampicilin) was inoculated and grown overnight (maximum of 16 h). Luria Bertani broth (1 liter, with added 37 µg/mL chloramphenicol and 100 µg/mL ampicilin) was inoculated with 10 mL of the overnight culture and grown at 37°C with shaking at 250 rpm until an optical density of 0.6–0.8 (A_600__nm_) was reached (∼3 h). Isopropyl-1-thio-b-D-galactopyranoside (1 mM final concentration) was added to induce expression. The flask was shaken for 3 h under the same conditions; cells were harvested by centrifugation (6000×g, 20 min) and washed once in 20 mM Tris-HCl, pH 8.0, before the cell pellet was frozen and stored until use. The frozen cell pellet was resuspended in 200 mL of 20 mM Tris-HCl, pH 8.0, and cells disrupted using a probe sonicator before collecting the inclusion bodies by centrifugation. Inclusion bodies were washed 4 times with 20 mM Tris-HCl, pH 8.0, and then once with 1% Triton X-100 in 20 mM Tris-HCl, pH 8.0. The remaining pellet was washed four more times with 20 mM Tris-HCl, pH 8.0, before solubilization in 25 mL of 20 mM Tris-HCl, pH 8.0, 6 M guanidinium hydrochloride, 10 mM dithiothreitol. The solubilized material was centrifuged for 30 min at 12,000×g and the supernatant (25 ml) diluted drop by drop into 4 liters of 20 mM Tris-HCl, pH 9.3, 0.3 M arginine monohydrochloride, 1 mM EDTA, 0.2 mM GSSG, and 1 mM GSH, pH 9.3. After 48 h at 4°C, under agitation, the liters were filtered (0.22 µm), and concentrated by ultrafiltration using a 10 kDa cut-off prep/scale TFF cartridge (Millipore) to approximately 300 mL. After centrifugation for 30 min at 12,000×g, the supernatant was concentrated in a 10 kDa membrane (regenerated cellulose, Millipore, MA) using a Amicon ultrafiltration device until a final volume of 30 mL. Samples were centrifuged again (12,000×g, 30 min), and then purified by HPLC as below.

### Protein purification

Agalectin (8 mL, ∼15 mg) was loaded into a HiPrep 16/60 Sephacryl S-100 HR (GE Healthcare) column equilibrated in 20 mM Tris-HCl, NaCL 0.15 M, pH 9.3 with a flow of 1 mL/min and connected to AKTA purifier system (Amersham Biosciences, Uppsala, Sweden) Pharmacia HPLC system. Protein was detected by peak absorbance at 280 and 220 nm, and fractions containing Agalectin (estimated by SDS/PAGE) were combined (4 mL), concentrated in centricon (10 kDa cut-off). Samples (∼8 mL) were loaded in a gel-filtration column (Superdex 75 HR10/30, Amersham Biotech) equilibrated in 20 mM Tris-HCl, 0.15 M NaCl, pH 9.3. Elution was carried out at flow rate of 1 mL/min and fractions containing Agalectin were combined. In order to add one more purification step, Agalectin was dialysed against Tris 1 mM, pH 9.3, and applied in a MONO-Q anion-exchange column (Pharmacia Biotech). Proteins were eluted with a linear gradient of NaCl (0–1 M) over 60 minutes at a flow rate of 0.5 mL/minute. Fractions containing Agalectin were combined and dialysed against TBS, pH 7.4.

### Agalectin sequence

Sequence similarity for Agalectin (gi 118795154) was performed using the BLAST program. No cleavage site predictions of the mature proteins used the SignalP program, in accordance with the unconventional secretion pathway of galectins [19]. The molar extinction coefficient (ε_280__nm_) of full-length Agalectin at 280 nm was obtained at http://www.expasy.ch/cgi-bin/protparam, yielding ε_280__nm_ = 32555 M–^1^.cm–^1^; A^0.1%^_280__nm/cm_ (1 mg/mL) = 1.723, molecular weight 18,894.0 (158 aa) and *pI* 4.96 (mature protein).

### SDS-PAGE and Edman degradation

Samples were treated with 4× NuPAGE lithium dodecyl sulfate sample buffer and 10× sample reducing reagent, then loaded in NuPAGE-Bis-Tris 4% to 12% gels with 2-(N-morpholino) ethanesulfonic acid (MES) running buffer (Invitrogen). Gels were Coomassie blue stained.

### Labeling of Agalectin

Agalectin was labeled with FITC using the Fluoreporter FITC protein labeling kit (F6434) from Invitrogen/Molecular Probes (Eugenes, Oregon, USA), as per manufacturer’s instructions.

### Glycan Microarrays

The Glycan-protein interaction resource (Department of Biochemistry, Emory School of Medicine) was developed by the Consortium for Functional Glycomics under the directions of Drs. David F. Smith, and Jamie Heimburg-Molinaro. Experiments were performed under the request protocol_3072. Agalectin was labeled with FITC as described by the manufacturer, and dialysed against binding buffer: 20 mM Tris, 150 mM NaCl, 2 mM Ca^2+^, 2 mM Mg^2+^. Then, 0.05% tween-20, and 1% BSA were added to the sample. Experiments were performed as described in detail at:

http://www.functionalglycomics.org/static/consortium/consortium.shtml.

### Fractionation of *An. gambiae* SGH

One hundred pairs of female *An. gambiae* SGH were dissected under the microscope, sonicated extensively, and centrifuged for 10 min at 13,000×*g*. The supernatant (∼100 µl) was collected and loaded in a Superdex G-75 10/300 GL gel-filtration column (GE healthcare Biosciences) equilibrated in PBS pH 7.4 containing 0.1% PEG 4000. Elution was carried out at 0.5 ml/min, with 0.5 ml fractions. Addition of PEG was critical in order to recover the hemagglutinating activity of the SGH. The fractions were subsequently tested for hemagglutinating activity as above. Control experiments demonstrated that the presence of PEG 0.1% did not affect the lectin properties of the SGH. In order to estimate the correct molecular weight of the candidates, the column was calibrated with Conalbumin (75 kDa), BSA (66 kDa), Ovalbumin (40 kDa), and Approtinin (6.5 kDa).

### Nano-Liquid Chromatography Mass Spectrometry (nano-LC MS/MS) of the purified fractions

A fraction containing the peak of lectin activity was separated by NU-PAGE 4–12% (MOPS buffer) and the gel was sliced in 20 bands, followed by tryptic digestion and nano-LC MS/MS essentially as described [20]. The MS results were matched against a database of *Anopheles gambiae* salivary gland transcriptome [7], [8].

### HL-60 cells culture and adhesion to P-selectin

P-selectin (50 µl, 2 µg/ml in PBS, 100 ng/well) (R&D systems, Minneapolis, MN) was immobilized overnight in Microfluor 2 Black “U” bottom 96 well microplate (#7205, Thermo Scientific), followed by washing 3 times in PBS. Blockade of non-specific binding sites was achieved with BSA (3% BSA, v/v), for one week. Human promyelocytic leukemia cells (HL-60 cells) were purchased from ATCC and grown in RPMI 1640 medium, containing 20% FBS following manufacturer’s instructions (ATCC, Manassas, VA). In the day of the experiment, cells were resuspended in RPMI, loaded with calcein-AM (2 µM in RPMI without BSA for 40 min, RT). Cells were centrifuged (1000 rpm, 10 min) and resuspended in RPMI containing 1 mM Ca^2+^ and 1 mM Mg^2+^ and 0.05% (v/v) Bovine Serum Albumin (BSA) (HBSS-Ca^2+^, Mg^2+^-BSA). Then, in an Eppendorf tube, HL-60 cells (in RPMI, Ca^2+^, Mg^2+^-BSA, at 2×10^6^/ml, final concentration 200,000 cells/well) were incubated for 15 min at RT with PBS, SGH or Agalectin (1 µM). Fifty l of this mixture was transferred to the wells (in quadruplicates). After 30 min at RT, plates were inverted and washed with 100 µL of RPMI, Ca^2+^, Mg^2+^-BSA. Negative controls were carried out in the presence of EDTA (10 mM) added to HL-60 cells. In some experiments, neutrophils (in HBSS) were used to substitute HL-60 cells. Fluorescence was determined at 490 nm/520 nm using a SpectraMAX Gemini XPS Fluorometer (Molecular Devices).

### Neutrophil isolation and labeling with Calcein-AM

Blood (7 ml, collected in 5 mM EDTA, final concentration) was carefully layered on 7-ml Mono-Poly Resolving Medium (1698049, MP Biomedicals, LLC), in 15-ml Falcon tues. The tubes were centrifuged at 863× *g* (2000 rpm, Sorval RT Legend) for 45 min at RT using acceleration and deceleration set at position 5. Two rings were observed; the upper ring was discarded, and the lower ring was collected and transferred to a 50-ml Falcon tube. Then, 40 ml HBSS were added, and cells were centrifuged again for 10 min at 1000 rpm. When contamination with red blood cells was visible, 1 ml of lysis buffer (cold Hepes 5 mM) was added to the tubes for 40 sec, followed by the addition of HBSS (45 ml) and centrifugation as above. The pellet was re-suspended in 5 ml HBSS with 0.05% BSA (v/v) and counted, and concentration was adjusted to 1.0×10^6^/ml. Cells were kept on ice. For some experiments, neutrophils (in HBSS without BSA) were labeled with Calcein-AM (2 µM) at RT for 40 min, under continuous rotation, centrifuged and re-suspended in HBSS buffer as above.

### Neutrophil adhesion to fibrinogen

This was performed as described with modifications [21]. Microfluor 2 black U-bottom microtiter polystyrene plates (Thermo Scientific, Waltham, MA USA) were coated overnight with human fibrinogen (50 µL, at 1 mg/mL estimated at 280 nm and adjusted for the ε_280__nm_ (0.1%, 1 mg/ml) = 1.51, in PBS, 50 µg/well). Wells were washed 3× with 200 µL PBS and blocked for 1 week with BSA 3% in PBS. Long term blockade with BSA was required to prevent non-specific binding of activated neutrophil to the plastic. In the day of the experiment, wells were washed with HBSS, 0.05% (v/v) BSA (without Ca^2+^ and Mg^2+^). Then, in an Eppendorf tube, HBSS (100 µl, supplemented with 2 mM Ca^2+^ and 2 mM Mg^2+^) and 100 µl neutrophils (final 0.5×10^6^/ml, or 100,000 cells) were combined, followed by addition of PBS or Agalectin (1 µM), and incubation for 15 min at RT. Fifty l of this mixture were added to the wells (in quadruplicates), followed by addition of 5 µl of fMLP diluted in HBSS-BSA 0.05% (500 nM, final concentration 50 nM), without plate agitation. After only 5 min at RT, plates were inverted and washed with 100 µL of HBSS, 0.05% BSA (without CaCl_2_ and MgCl_2_). Negative controls were carried out in the presence of EDTA (10 mM) added to neutrophils, or without addition of *f*MLP. Fluorescence was determined after every washing at 490 nm/520 nm using a SpectraMAX Gemini XPS Fluorometer (Molecular Devices).

### Microscopy

In some experiments, cells previously incubate with SGH were visualized for detection of clumps, using Zeiss Axiovet 25 microscope (Carl Zeiss, Inc.; Thornwood, NY).

### Endothelial cell adhesion to matrix proteins, coagulation tests, and platelet aggregation assays

These experiments were performed essentially as described [22], [23].

### Modeling

The galectin was modeled using the Phyre2 server and SwissModel with the galectin domain of porcine adenovirus type 4 nadc-1 isolate2 (PDB accession code 2WSU) as a homology template. The figure was produced using Pymol. CLUSTAL alignments were performed at http://npsa-pbil.ibcp.fr.

### Statistical analysis

Results are expressed as mean ± S.E. (GraphPad 6.0 Software, Inc., San Diego, CA). Statistical significance was set at *p*<0.05 (*t* test or analysis of variance).

## Results and Discussion

### An. gambiae salivary gland homogenate displays hemagglutinating activity

**Figure 1:**
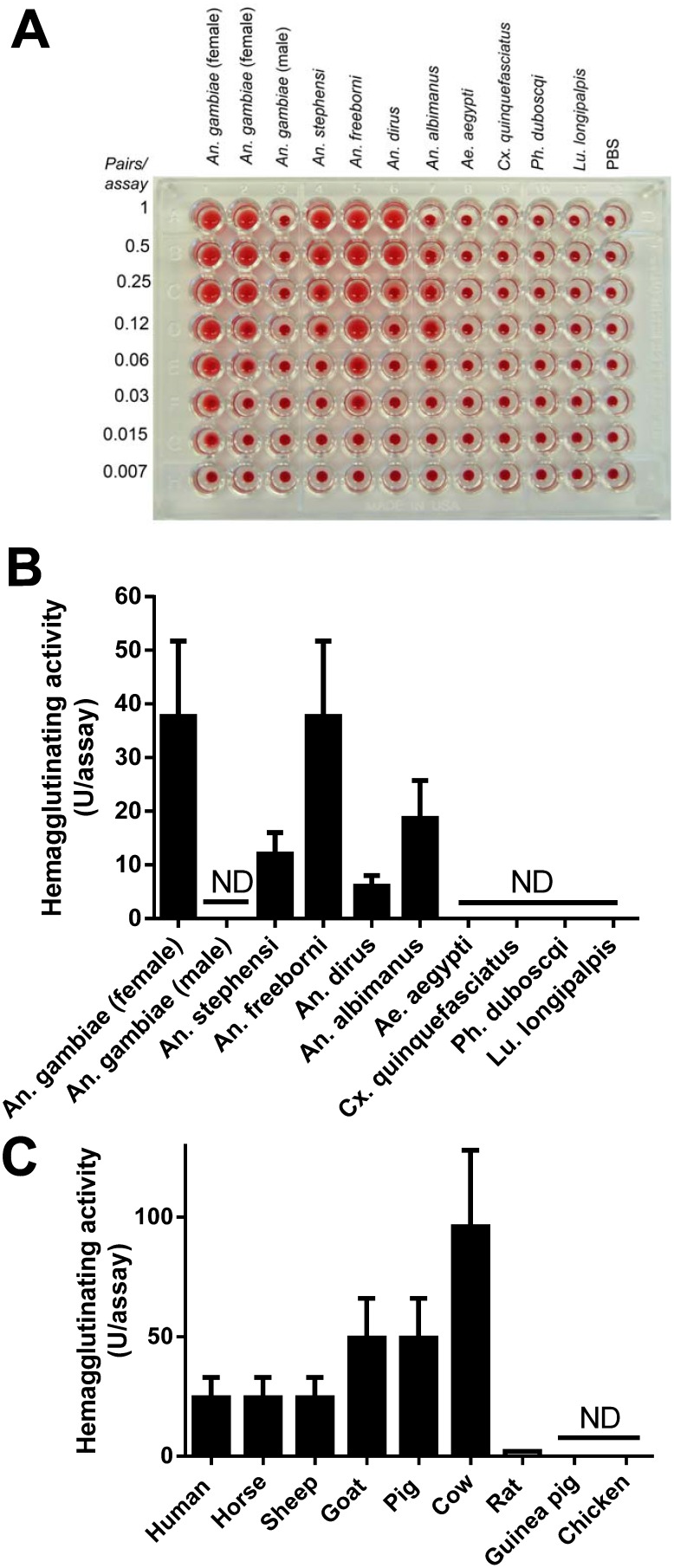
Lectin activity for the SGH of Anophelinae mosquitoes, but not for other genus. (A) SGH from *An. gambiae* (male and female), *An. stephensi, An. freeborni, An. dirus, An. albimanus, Ae. aegypti, Culex quinquefasciatus, Phlebotomus duboscqi, Lutzomyia longipalpis,* were serially diluted followed by addition of O-type RBC. Two preparations of female *An. gambiae* SGH were tested. (B) Quantification of lectin activity. U/assay was given as 1/*n*, where *n* is the maximum dilution promoting agglutination presented in (A) ND, not detected. (C) Species-specificity. SGH of *An. gambiae* was tested in RBC from human, horse, sheep, goat, pig, cow, rat guinea pig, and chicken.

Lectin activity in the salivary gland of mosquitoes has been reported before [12]. However, the functional and molecular characterization of the molecule(s) involved in these interactions, as well as its sugar specificity has not been determined. To gain further information, the SGH from several mosquitoes were dissected and tested for agglutination of RBC (hemagglutinating activity). Figure 1A shows a typical result where the SGH was serially diluted and RBC added subsequently. Figure 1B shows the quantification of the results estimated as 1/*n*, where *n* is maximum dilution of the SGH that promotes agglutination. The following specific activities (U/assay) were found: *An. gambiae* (female, 32 U/assay), *An. stephensi* (8 U/assay), *An. freeborni* (32 U/ml), *An. dirus* (4 U/ml), *An. albimanus* (8 U/ml). No activity was detected for *An. gambiae* male, *Aedes aegypti*, *Culex quinquefasciatus*, *Ph. duboscqi*, and *Lu. longipalpis*. PBS was used as a negative control. Figure 1C shows that *An. gambiae* SGH promotes agglutination of erythrocytes from humans (type O, ∼25 U/assay), horse (∼25 U/assay), sheep (∼25 U/assay), goat (∼50 U/assay), pig (∼50 U/assay), cow (∼100 U/assay), and to a lesser extent from rats (∼2U/assay). No activity was observed when erythrocytes from guinea pigs or chickens were used in the assay. Identical results were obtained when the hemagglutinating activity of *An. gambiae* SGH was performed in the presence of CaCl_2_ (5 mM).

### Sialylated polysaccharides inhibit lectin activity from the SGH of An. gambiae

The specificity of the agglutinating activity of the SGH of *An. gambiae* was tested by using sulfated polysaccharides, matrix proteins, sialylated molecules, sialic acid containing oligosaccharides, gangliosides, and simple sugars (Table 1). Among the sulfated polysaccharides, dextran sulfate (DS) 500,000 was the most potent inhibitor of agglutination, followed by DS 50,000 with an inhibitory concentration (IC) of 3.715 and 37.5 pM, respectively. No residual activity was found for non-sulfated dextran indicating that sulfate moiety is critical for inhibition of the lectin activity of the SGH. Fucoidan, which is an algae sulfated polysaccharide inhibited agglutination with IC 470 pM. Heparin Grade II was found to be a good inhibitor of agglutination with an IC ∼78 µg/ml. On the other hand, no inhibition was found for chondroitin sulfate A, chondroitin sulfate C, heparan sulfate, and keratan sufate. Next, sulfated proteins, and other selected molecules were tested in the assays. Laminin and heparin sulfate proteoglycan (HSPG) inhibit agglutination with IC below 100 nM. Glycophorin was not effective at 0.5 mg/ml (maximum tested concentration), and asialofetuin, orosomucoid, and fetuin (up to 5 mg/ml) were not inhibitory.

**Table 1:** Specificity of *An. gambiae* SGH hemagglutinating activity.

Ligand	Inhibitory concentration*
**1. Sulfated polysacharides**	
Dextran sulfate 500,000	<3.715 pM
Dextran sulfate 50,000	<37.15 pM
Fucoidan	470 pM
Heparin Grade II	78 µg/ml
Condrotin sulfate A	NI up to 1.25 mM#
Condrotin sulfate C	NI up to 1.25 mM
Heparan sulfate	NI up to 0.25 mM
Keratan sulfate	NI up to 0.25 mM
Dextran 500,000	NI up to 125 µM
Dextran sulfate 5,000	NI up to 1.25 mM
**2. Proteins**	
Laminin	18 nM
Heparan Sulphate Proteoglycan	93 nM
Glycophorin	NI up to 0.5 mg/ml
Asialoglycophorin	NI up to 5 mg/ml
Orosomucoid	NI up to 5 mg/ml
Fetuin	NI up to 5 mg/ml
**3. Sialic acid-containing glycans**	
Sialic acid	6.25 mM
3′-sialyl Lewis X	0.3125 mM
3′-sialyl Lewis A	NI up to 1.25 mM
3′-sulfated Lewis A	0.625 mM
3′-sialyl lactose	0.156 mM
6′-sialyl lactose	0.078 mM
Tetrasacharide A	NI up to 2.5 mM
Tetrasacharide B	NI up to 2.5 mM
Tetrasacharide C	NI up to 2.5 mM
Di-sialyl lactose-tetraose	NI up to 2.5 mM
3′-sialyl fucosil lactose	NI up to 2.5 mM
**4. Gangliosides**	
Monosialoganglioside (GM3)	0.312 mM
Disialoganglioside (GD1a)	0.312 mM
Disialoganglioside (GD1b)	0.625 mM
Trisialoganglioside (GT1B)	0.156 mM
Tetrasialoganglioside (GM1)	0.625 mM
Tetrasialoganglioside (GQ1B)	0.2 mM
**5. Simple sugars**	
Maltose	NI up to 25 mM
Galactose	NI up to 25 mM
Trehalose	NI up to 25 mM
Manose	NI up to 25 mM
Glicose	NI up to 25 mM
N-acetil-galactosamine	NI up to 25 mM

*Inhibitory concentration; minimal concentration promoting inhibition of agglutination.

# NI; non-inhibitory at the maximum tested concentration.

We also verified the inhibition of hemagglutination by sialic acid-containing oligosaccharides. Table 1 shows that sialic acid inhibited the activity of the SGH at 6.25 mM. Other sialyl-containing proteins such as 3′sialyl Lewis X, 3′-sulfated Lewis A, 3′- sialyl lactose and 6′-sialyl lactose were active with IC varying from 0.156–0.625 mM. No activity was observed for tetrasacharides A, B, and C, or di-sialyl-lacto-tetraose, or 3′-sialyl fucosil lactose. Table 1 also shows that several gangliosides, including GM3, GD1a, GD1b, GT1B, GM1, and GQ1B were effective inhibitors of agglutination promoted by the SGH, with MIC ∼0.3 mM. Finally, simple sugars (maltose, galactose, trehalose, manose, glucose, and N-acetyl-galactosamine) did not block agglutinating activity of the SGH at 25 mM (maximum tested final concentration).

### SGH of An. gambiae affects the interaction of HL-60 cells to immobilized P-selectin

**Figure 2:**
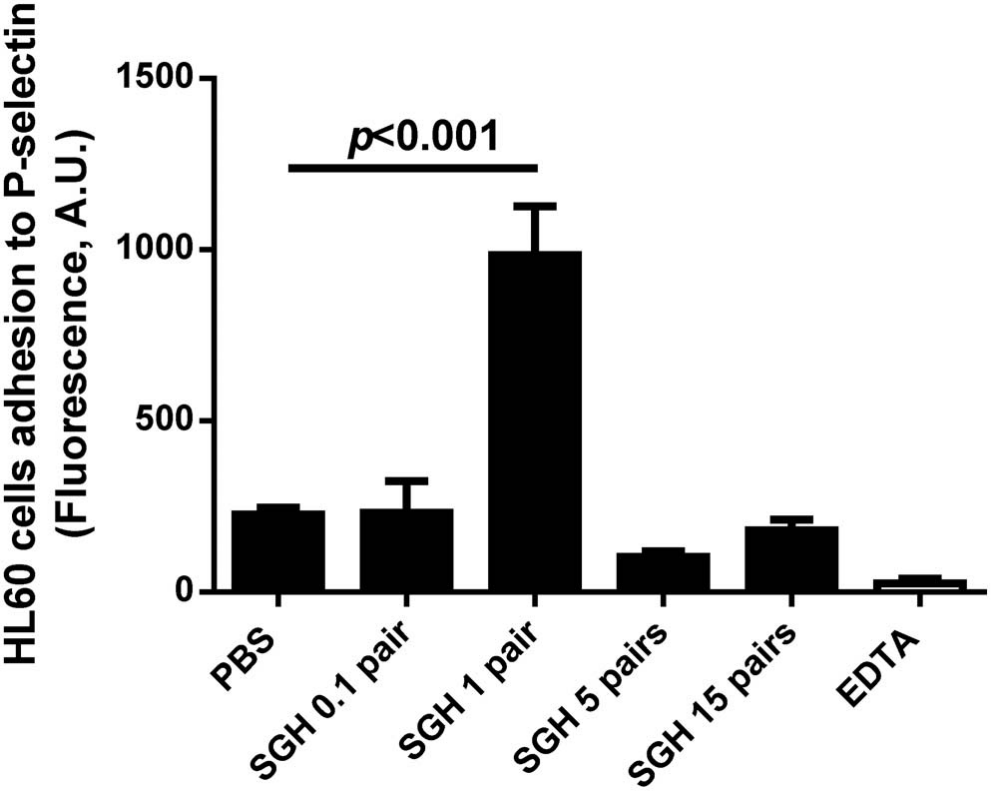
*An gambiae* SGH increases HL-60 cell adhesion to immobilized P-selectin. Calcein-labeled HL-60 cells were incubated with SGH (0–15 pairs/assay), and added to the plates containing immobilized P-selectin. After 30 min, cells were washed and adhesion was estimated by fluorescence. Results are the mean±SD of triplicate determinations (p<0.05, ANOVA).

The finding that the lectin activity of SGH of *An. gambiae* is inhibited by sialylated molecules suggested that it may interfere with the function of neutrophils which express P-selectin glycoprotein ligand-1 (PSGL-1), a receptor rich in sialyl Lewis X. PSGL-1 interacts with P-selectin present in the endothelial cells [24]. In an attempt to verify this hypothesis, HL-60 cells were grown in culture, and incubated with *An. gambiae* SGH. Visualization under the microscope showed that HL-60 cells formed clumps indicative of agglutination (not shown). In order to verify how this interaction might affect the interaction of neutrophils and endothelial cells, HL-60 cells were incubated with SGH and added to wells containing immobilized P-selectin. Figure 2 shows that *An. gambiae* SGH increases the adhesion of HL-60 cells to immobilized P-selectin, at 1–5 pairs/assay, but at higher concentrations the effect was no longer observed. Our interpretation is that agglutination of the cells at certain concentrations of SGH facilitates the adhesion of large clumps to P-selectin. However, at >5 pairs/assay, excessive clumping of HL-60 cells promotes loosely adherent HL-60 cells, facilitating detachment during the washing steps.

### Cloning and expression of An. gambiae putative lectin

In an attempt to identify the molecule with lectin activity, we analyzed the transcriptome of *An. gambiae* salivary gland [7], [8]. It exhibits one member of the galectin family [25], herein named Agalectin, and one member of the C-type family of proteins [26], herein named Anophelectin [7], [8].

### Characterization of Agalectin

**Figure 3:**
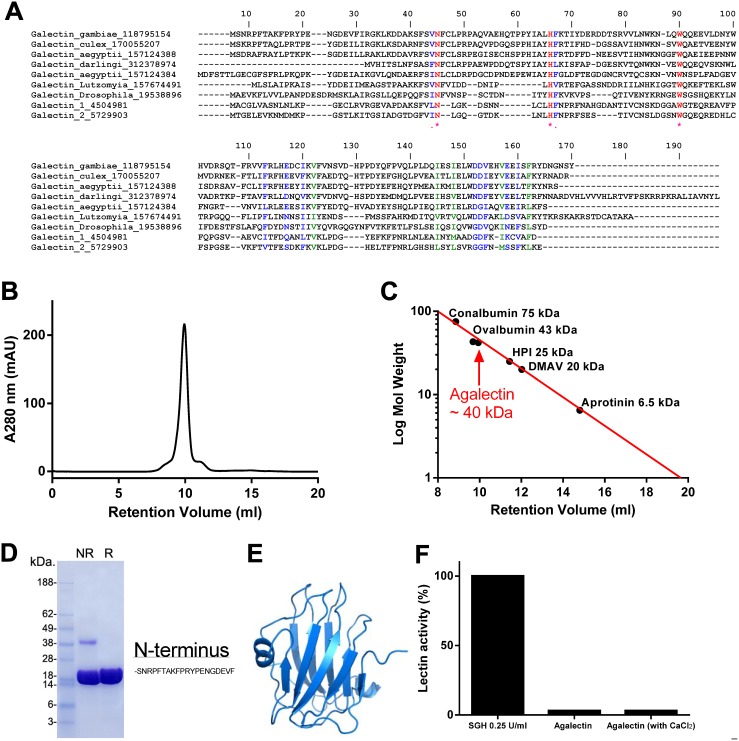
Expression of Agalectin, a member of the galectin family of lectin. (A) Clustal aligment of Agalectin with orthologs from Diptera, human galectin-1 and galectin-2. (B) Galectin purification. The chromatogram of the last purification step is shown. (C) SDS-PAGE of Agalectin, under reducing, or denaturing conditions. (D) Molecular weight determination. The retention volume of Agalectin was compared with molecular weight markers, and determined to behave as a dimer in solution. (E) Molecular modeling of Agalectin. Based on the galectin domain of porcine adenovirus (PDB 2WSU). (F) Lectin activity. Agalectin does not agglutinate RBC, with or without CaCl_2_ (n = 3).

Galectins are lectins that bind β-galactosides such as lactose and N-acetylactosamine, in free form or contained in glycoproteins or glycolipids. They are located intracelularly and extracelularly. Extracellular galectins interact with glycans in the cell surface and induce various celullar responses, including production of cytokines, cell adhesion, migration and apoptosis [25], [27]. Agalectin is expressed 6 times more in female than in male SGH and is highly enriched in the salivary gland in comparison to the carcasses and adult males [8]. Figure 3A shows the alignment of Agalectin with galectins from several other organisms including *Culex quinquefasciatus*, *Ae.aegypti*, *An. darlingi*, and *Lu. longipalpis*, *Drosophila melanogaster*, and human galectin-1 and -2. Because some galectin members recognize sialylated glycans [28], and could account for the observed hemagglutinating activity of the gland, the cDNA sequence coding for mature Agalectin (gi 118795154) was synthesized and cloned in a Pet17b vector, followed by expression and refolding as reported in Methods. Analysis of the sequence indicates that it contains no signal peptide, a molecular weight of approximately 20 kDa, *pI* 4.96 and 2 cysteines.

Figure 3B shows the purification of recombinant Agalectin in gel-filtration, where it elutes as a peak at ∼10 ml retention volume. Calibration of the column with known molecular weight markers indicate that the apparent molecular weight corresponds to a ∼40 kD molecule in solution (Figure 3C). Therefore, Agalectin behaves as a dimer. In SDS PAGE, Agalectin migrates as a 20 kDa protein, under denaturing, or denaturing and reducing conditions, indicating that it does not form a covalent homodimer (Figure 3D). The N-terminus SNRPFTAKPRYPENGDEVF was confirmed by Edman degradation. Figure 3E depicts a model for Agalectin showing that it exhibits most features that characterizes the galectin structure of porcine adenovirus (PDB 2WSU). Our results also demonstrate that Agalectin (1 µM) did not induce agglutination in the absence or presence of 5 mM CaCl_2_ while it was observed with SGH of *An.gambiae* (Figure 3F). Agalectin (1 µM) also did not affect neutrophil adhesion to immobilized P-selectin or to fibrinogen (not shown), excluding interaction with sialyl Lewis X. We also tested the sugar binding properties of FITC-Agalectin using a Glycan arrays. No interaction was found for more than 500 sugars, listed at http://www.functionalglycomics.org/static/consortium/consortium.shtml.

Different sequences have been deposited in the Genebank for Agalectin. These sequences are almost identical with the exception of few residues in the C-terminus, which represent part of the gene where confidence for sequencing is not optimal. Therefore, we also expressed a variant of Agalectin (gi 347965476), which displays a cysteine as the last aminoacid in the C-terminus. This variant displayed similar chromatographic behavior as gi 118795154 and it was also devoid of hemagglutinating activity, with or without CaCl_2_ (not shown).

Because galectins [25], [27], [29] affect acute and chronic inflammation through a number of mechanisms, we tested it in different screening assays. Agalectin (up to 1 µM) did not induce nor inhibit platelet aggregation by collagen, nor affected blood coagulation and endothelial cell adhesion to fibronectin, or laminin (not shown, n = 3). The function of this protein remains unknown. We cannot exclude that Agalectin is an intracellular protein modulating signaling pathway in the salivary gland, or that it displays housekeeping properties.

### Anophelectin

A second candidate for the agglutinating activity of the SGH of *An. gambiae* is Anophelectin (gi 58388509), a member of C-type lectins [26]. Anophelectin is a putatively secreted molecule based on the Signal P results. CLUSTAL alignment indicates that it exhibits residues highly conserved in other members of the C-type lectin family members (not shown). Since the level of expression of this lectin is only 1.5 times higher in female than in males [8], it is unlikely that it explains why SGH from female exhibits lectin activity and males do not.

### Purification of the lectin from the SGH of An. gambiae

**Figure 4:**
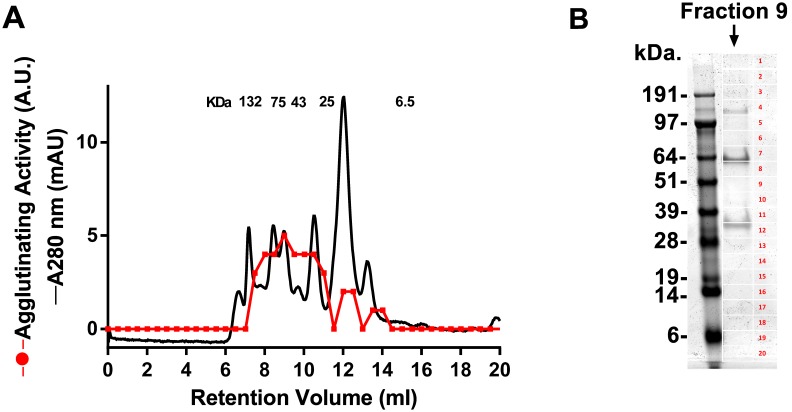
*An. gambiae* SGH purification and MS analysis. (A) One hundred pairs of SGH were loaded in a gel-filtration column, and eluted in PBS containing 0.1% PEG 4000. The fractions were tested for lectin activity. (B) MS analysis. The fraction (retention volume at 9 ml) containing the highest activity was used for 1D PAGE. Twenty gel bands were cut, followed by in-gel tryptic digestion, and nano-LC MS/MS (results presented in Table S1).

To identify the molecule with lectin activity, SGH obtained from 100 pairs of *An. gambiae* was loaded in a gel-filtration column, and eluted with PBS. All fractions were negative for lectin activity suggesting that the active molecule was lost by adsorption. To avoid this limitation, the column was equilibrated in PBS containing 0.1% PEG 4000, and the SGH obtained from additional 100 pairs was loaded in the column. Control experiments indicated that PEG 4000 did not affect the lectin activity of the SGH. Fractions were tested for lectin activity as described above. Figure 4A shows the elution profile of the SGH where several peaks were observed at different retention volumes. Activity was found spread in several fractions with a major peak of activity at approximately 75 kDa.

### Mass spectrometry of the active fraction

The “sticky” nature of the molecule with lectin activity from the SGH, and the relative loss of activity after gel-filtration, in addition to the sensitivity to organic solvent, precluded the use of additional chromatography steps for purification. In order to characterize the protein present in the fraction corresponding to the peak of activity after gel-filtration, fraction 9 (corresponding to retention volume 9 ml) was loaded in a 4–12% NU-PAGE gel (Figure 4B). Twenty bands were sliced, followed by in-gel tryptic digestion, and nano-LC MS/MS. Results were blasted against a transcriptome database [8], and annotated in Table S1. While several proteins were identified, none of them matched sequences of C-type lectin or galectin families.

## Concluding Remarks

Our results determined for the first time that *An. gambiae* hemagglutinating activity is specific for sulfated glycans. Such specificity is important because different cell types and molecules display sialylated glycoconjugates and might modulate parasite-vector-host interactions, or mosquito physiology. For example, a lectin targeting sulfated molecule may interfere with the function of sialyl Lewis X-containing PSGL-1 which mediates the interaction of neutrophils with P-selectin expressed by endothelial cells. This interaction may interfere with inflammation. Interestingly, a high molecular weight component from the SGH of *An. stephensi* exhibits neutrophil chemotactic activity and binds to lentil-sepharose column [30]. Bite by *Anopheles* sp is also associated with inflammatory events characterized by fluid extravasation, neutrophil influx by a mechanism involving dermal mast cell degranulation [31]. However, it remains to be determined whether these activities are blocked by sulfated glycans. In the vector side, salivary glands contain several glycoconjugates in the surface which are critical for recognition of sporozoites, a step crucial for invasion of the glands [32]–[34] and for parasite maturation [35]. Alternatively, salivary lectins may be ingested with blood and in the midgut may contribute to agglutinate RBC, facilitating digestive processes [36]–[40].

Finally, while the molecular identity of the sulfated glycan-specific lectin remains to be determined, our MS results provide a database which serve as a starting point to identify this protein, and eventually, if and how it mediates parasite-vector-host interactions.

## Supporting Information

Table S1 Results for in-gel tryptic digestion, and nano-LC MS/MS of PAGE presented in Figure 4B.
(XLSX)
